# Critical Knowledge Gaps in Our Understanding of Environmental Cycling and Transmission of Leptospira spp.

**DOI:** 10.1128/AEM.01190-17

**Published:** 2017-09-15

**Authors:** Veronica Barragan, Sonora Olivas, Paul Keim, Talima Pearson

**Affiliations:** a The Pathogen and Microbiome Institute, Northern Arizona University, Flagstaff, Arizona, USA; b Instituto de Microbiologia, Colegio de Ciencias Biologicas y Ambientales, Universidad San Francisco de Quito, Quito, Ecuador; Rutgers, The State University of New Jersey

**Keywords:** Leptospira, leptospirosis, environment, water, soil, transmission, survival

## Abstract

Exposure to soil or water contaminated with the urine of Leptospira-infected animals is the most common way in which humans contract leptospirosis. Entire populations can be at high risk of leptospirosis while working in inundated fields, when engaging in aquatic sports, or after periods of heavy rainfall. The risk of infection after contact with these environmental sources depends on the ability of Leptospira bacteria to survive, persist, and infect new hosts. Multiple variables such as soil and water pH, temperature, and even environmental microbial communities are likely to shape the environmental conditions needed by the pathogen to persist. Here we review what is known about the environmental phase of the infectious Leptospira transmission cycle and identify knowledge gaps that will serve as a guide for future research.

## INTRODUCTION

Leptospirosis is a zoonotic disease caused by spirochete bacteria in the genus Leptospira. It produces different symptoms and signs, such as headaches, fever, jaundice, kidney and liver failure, and even death (1, 2), in 1.03 million annual cases around the world. Leptospira bacteria are shed in bodily fluids (urine, placenta, and vaginal fluids), and infection occurs when the pathogen penetrates the skin through small abrasions or mucosal membranes (e.g., the eyes and mouth) (1, 3). Veterinarians, farmers, and other individuals who work with animals and their products are at high risk of contracting the disease because of the likelihood of exposure to contaminated fluids (1). Other occupations (e.g., plumbing, sewer work, and garbage collection) also involve indirect contact with infected animal products such as urine. Likewise, agricultural workers may be exposed to contaminated soil or water. Importantly, when the pathogen is shed into the environment, nonoccupational exposure in urban and rural settings is also possible and can place entire populations at risk for leptospirosis (2). Because of the complex and diverse interactions between animals (including humans) and their living environment, the risk of infection by exposure to contaminated environmental sources is not well understood and is thus challenging to control. Our aims here are to provide a broad overview of environmental survival, persistence, and transmission of infectious Leptospira bacteria and to identify knowledge gaps to guide future research.

## EXPOSURE TO SOIL OR WATER IS A COMMON RISK FACTOR FOR HUMAN LEPTOSPIROSIS

The literature is replete with examples of human leptospirosis cases without clear evidence of direct contact with animals that are thus likely due to contact with contaminated soil or water. Such examples are common among farmers working in inundated fields or among fishermen, with mean odds ratios (ORs) of >2 (1, 4). Risk factors include exposure to stagnant or moving water or mud (1, 5–7). Manual laborers such as trash collectors, miners, or cane cutters are particularly prone to skin cuts and abrasions that may put them at additional risk for contracting leptospirosis. Recreational exposure has also been widely associated with this disease, with mean ORs similar to those related to occupational activities, particularly after swimming and practicing aquatic sports in rivers and other water sources. Outbreaks have been linked to kayaking, surfing, canoeing, rafting, triathlons, and military training exercises, among others with documented exposure to water (2). In addition to occupational and recreational activities, weather conditions have also been associated with Leptospira infection. Increased leptospirosis rates are usually reported during and after extreme weather events or heavy rainfall, especially in tropical countries. Moreover, the risk of infection due to exposure to floodwater, mud, or wet soil associated with these events varies from region to region and in some cases shows seasonal variation (7–11).

While the association of leptospirosis with rain and extreme weather events is well established, we can only speculate about how these conditions might favor the persistence and dispersal of Leptospira bacteria in the environment in a manner that leads to increased human infections. Site-specific features may further impact the survival and dispersal of this pathogen. In urban settings, large quantities of floodwater frequently overwhelm sewage systems, increasing the risk of infection through direct contact with contaminated water or by facilitating dispersal to soils that may be primed by rainfall to become increasingly suitable for pathogen survival and persistence. An additional consequence of flooding is that rats and domestic animals may be forced to seek refuge in the same noninundated places as humans, increasing the likelihood that noninfected animals (and humans) will be exposed to and have contact with infected animals (12). In rural settings, an increased quantity and diversity of peridomestic animals, which may be particularly important sources of Leptospira bacteria (13), may increase transmission in this manner. Moreover, under flood conditions where dry habitat is scarce, sylvatic animals may increasingly encroach on the peridomestic environment, further complicating the environmental cycling of infectious Leptospira bacteria.

## RESERVOIRS AND THE ENVIRONMENTAL LOAD OF INFECTIOUS LEPTOSPIRA

The risk of human infection posed by different animal species is likely to be greatly influenced by the amount of Leptospira bacteria shed into the environment and the likelihood of human contact with the resulting contaminated soil. A wide diversity of peridomestic animals (rats, horses, cows, dogs, and pigs) and feral animals (bats, coyotes, sea lions, and even frogs) can carry Leptospira bacteria in their kidneys and therefore presumably excrete the pathogen into the environment. The amount of pathogen that these animals shed is likely to be very important for the establishment of environmental sources and the risk of infection upon exposure to those sources. Rats shed about 5.7 × 10^6^ Leptospira bacteria/ml of urine; and cows, deer, dogs, mice, and humans have been reported to shed an average of 3.7 × 10^4^, 1.7 × 10^5^, 1.4 × 10^2^, 3.1 × 10^3^, and 7.9 × 10^2^ Leptospira bacteria/ml of urine, respectively. Information on the quantities of Leptospira bacteria shed by other animals is completely lacking (14). Host weight might also affect the amount of pathogen excreted, as has been reported for naturally infected young black rats, where higher weight was significantly associated with renal Leptospira loads (15). Multiple variables such as the volume of urine shed by each host, prevalence among hosts, and local host densities will define the pathogen load excreted into an environment (13, 14, 16, 17). In urban areas where small animals (rats and dogs) predominate, a high risk of human infection may depend on a high animal densities and a high prevalence of disease among these animals. Conversely, in places where large mammals (e.g., cows) are the main reservoirs, the sheer volume of urine excreted into the environment by very few animals may convey vast amounts of Leptospira bacteria and result in a high risk of human infection (13, 14). Also, Leptospira bacteria shed by domestic and peridomestic animals may present a greater infection risk to humans than those presumably shed by wild animals in areas not frequently visited by humans. The roles of different animals in transmission among animals and environmental maintenance of Leptospira bacteria have not been investigated.

## INOCULUM REQUIRED FOR INFECTION

Leptospira infection typically occurs when bacterial spirochetes penetrate the body through mucosal membranes or skin cuts (1, 2), enter the bloodstream of the host, and disseminate to cause systemic infection (3, 18). Unfortunately, our knowledge of the quantity of Leptospira bacteria required to cause infections in a natural environment is based on laboratory studies using hamster (19, 20), guinea pig (21, 22), and monkey (23) models. Animals inoculated with large doses of Leptospira bacteria through the conjunctival (10^5^ to 10^8^ bacteria) (22, 24, 25), subcutaneous (2 × 10^6^ bacteria) (26), and epicutaneous (5 × 10^8^ bacteria) (27) routes have been used to establish infections. However, these studies were not intended to provide infectious dose estimates and it is likely that infectious doses are considerably smaller because such extremely large doses are very unlikely to occur in nature. The greatest reported load of Leptospira bacteria in an environmental sample was 10^4^ Leptospira bacteria/ml (28, 29). Even direct sampling of urine from animals is likely to results in an amount of Leptospira bacteria that is less than what is typically used to induce laboratory infections; median amounts of Leptospira bacteria per milliliter of urine from infected animals range from 10^2^ (dogs) to 10^6^ (rats) (14). Swallowing water is thought to be an important route of entry (2); however, we were not able to find any published study that used the oral or intranasal inoculation route to establish infection. The relationship between the infectious dose and route of infection with different strains is a basic but critical knowledge gap that is crucial for accurately defining the risk of infection in animals (and humans) after contact with contaminated environmental sources.

## CURRENT KNOWLEDGE OF PERSISTENCE OF INFECTIOUS LEPTOSPIRA IN THE ENVIRONMENT

The survival and longevity of a pathogen once it is shed into the environment will have a direct bearing on the infection risk. Although few rigorous studies have considered the environmental phase of infectious Leptospira bacteria, relevant observations about their presence and survival have been compiled since the beginning of the 20th century (30) and suggest that environmental survival and persistence are highly dependent on environmental conditions. Such conditions include the medium type, as well as the location and seasonal variation. Also, even though the survival and persistence rates of these bacteria may differ among species and strains, comparative experiments have not been performed. There is therefore a great need for research to address how multiple biotic and abiotic variables interact together and with different pathogen strains to influence environmental survival and the ability to infect another host.

### Presence of infectious Leptospira in the natural environment.

In the last decades, the presence of infectious Leptospira bacteria in different natural environments has been evaluated by culturing the pathogen or by detecting its DNA. Most of these reports are observational but have shaped our understanding of environmental conditions and media likely to harbor these pathogens. Leptospira bacteria have been found in water and soil samples from rural and urban settings (31–33), in the jungle (34), after periods of heavy rainfall (35), and even during the summer (36). In summary, Leptospira bacteria have been found in a wide variety of environments, but unfortunately, only a few systematic studies provide detail information on the positivity (prevalence) and sources of the samples analyzed (Table 1) (17, 28, 29, 32, 33, 37–52). Results from individual studies suggest that the likelihood of finding infectious Leptospira bacteria in the environment is site dependent, differing among regions, medium sources, and seasons. For example, analysis of water samples collected in South Andaman Island showed higher positivity in urban sewage water and household drainage water than in ponds or civic toilet drainage. In the same study, higher positivity was found in rural paddy field water than in streams, ponds, and other water sources (39). Likewise, samples collected in streams in the Peruvian Amazon Basin show higher positivity than well water samples and there were higher pathogen concentrations in samples collected in urban settings than in those from rural ones (28). An important variable that might impact the likelihood of contact with Leptospira bacteria in the environment is animal urine dilution. We expect large amounts of pathogens washed into small water bodies such as stagnant water or ponds formed after periods of heavy rainfall because of the relatively low dilution of urine (53, 54), in contrast with big water bodies such as rivers or floodwater, where we expect highly diluted urine and lower pathogen concentrations. We can speculate that Leptospira aggregation behavior (55) might counteract the dilution factor in big water bodies, maintaining concentrations sufficient for infection. Such aggregation might partially explain why many studies do not detect infectious Leptospira bacteria in large bodies of water in regions where leptospirosis is endemic or when investigating outbreaks (56, 57). Detection of infectious Leptospira bacteria in the environment is also subject to seasonal variation (17). This might explain why the pathogen is not always found in environmental samples collected from localities where leptospirosis is endemic, such as Kelantan, Malaysia (Table 1) (38). These and other studies that have found infectious Leptospira bacteria (or their DNA) in the environment provide valuable epidemiological information about the general site characteristics that influence pathogen presence. Specific abiotic and biotic microsite conditions may also impact the presence of Leptospira bacteria in soil and water.

**TABLE 1:** Studies with detailed data on the positivity of infectious Leptospira bacteria in environmental samples

Sample type and origin	Reference(s)	% (no.) of samples positive		Source(s) of positive samples^*a*^	Leptospira species	Detection/identification method(s)
		Rural	Urban			
Water						
South Asia	39	9 (133)	12.4 (113)	Urban household drainage and sewage, rural paddy fields and water from stream and vegetable fields	Pathogenic and intermediate	Culture and PCR/PCR and sequencing
	40	22.1 (86)	26.8 (56)	Urban sewers; rural sewers, rainwater, and paddy fields	Pathogenic	PCR
Southeast Asia	38	0 (36)	0 (36)			Culture/PCR and sequencing
	31		6.4 (110)	Urban floodwater	Pathogenic and intermediate	Culture and PCR/PCR and sequencing
	41	0 (18)	8.3 (12)	Urban floodwater	Pathogenic	PCR
	42		2.5 (121)	Urban street drain water and lake water	Pathogenic and intermediate	Culture/PCR and sequencing
	43	5.5 (18)	0 (39)	Rural water	Intermediate	Culture/PCR and sequencing
	85	21.4 (14)		Rural underground water	L. interrogans, *L. weilii*	PCR/sequencing
	37		1.9 (324)	Urban stagnant water	L. borgpetersenii, L. interrogans, *L. wolffii*, *L. inadai*	Culture/PCR and sequencing
High-income Asia Pacific	43		6.3 (16)	Urban water from a university campus	*L*. alstonii	Culture/PCR and sequencing
Caribbean	44	18.2 (44)		Rural puddles, mountain springs, and water dams	Pathogenic	PCR
Central Latin America	33	3.7 (54)		Rural water from farms	Pathogenic	Culture/PCR
	45	3.7 (54)		Rural farm wastewater	L. interrogans	Culture/PCR
Andean Latin America	28	25.4 (236)	47.4 (192)	Urban gutters, river shore, puddles, and underground water; rural stream and well water	Pathogenic and intermediate	Culture and PCR/PCR
Southern Latin America	32	13.5 (570)		Rural wells, barrels, old tires, ponds, rivers, canals, and springs	Pathogenic	PCR
	17	19.6 (576)	16.7 (240)	Urban puddle, human drinking water, flowing source, container; rural animal drinking water, puddle, flowing source, container	L. interrogans, *L*. kirschneri, *L. weilii*, pathogenic	PCR/PCR and sequencing
Tropical Latin America	46		1 (100)	Urban community water supply	Pathogenic	PCR
Western Europe	29		6.4 (47)	Urban ponds	Pathogenic	PCR
	47		50 (4)	Urban water from toilet	L. interrogans	PCR/PCR and sequencing
	48		3.9 (151)	Urban river, canal water	Pathogenic	PCR
Central Europe	49	1.9 (104)		Rural wells	Pathogenic	PCR
High-income North America	50	100 (22)		Rural and urban stream water	Pathogenic	PCR/sequencing
	51	50 (2)		Rural stagnant pool	Pathogenic	Culture/serotyping
Soil						
Southeast Asia	38	0 (36)	2.8 (36)	Urban market soil samples	*L*. alstonii	Culture/PCR
	42		0 (30)			
	43	0 (3)				
	43		47.8 (23)	Urban inundated soil	Pathogenic, *L. kmetyi*	Culture/PCR
	37		11.6 (292)	Urban soil	*L. noguchii*, L. borgpetersenii, *L. weilii*, *L. wolffii*	Culture/PCR and sequencing
High-income Asia Pacific	43		25 (12)	Urban soil	*L* alstonii	Culture/PCR and sequencing
Western Europe	47		100 (2)	Urban soil from inside and outside a pond	L. interrogans	PCR/PCR and sequencing
Central Europe	49		0 (108)			
Eastern Europe	52		0.8 (630)	Urban soil near a lake	Pathogenic	Culture/serotyping

a Rural areas include rural villages and farms.

### Abiotic conditions impacting pathogen survival in the environment.

High morbidity in tropical climate regions, especially during periods of heavy rain, and the sporadic nature of cases that typify temperate regions (58) are consistent with the ability of Leptospira bacteria to better survive and persist under specific environmental conditions. Survival and persistence of infectious Leptospira bacteria in the environment may rely on the interaction of multiple factors, including abiotic soil and water conditions. These pathogenic spirochetes can live in soil with a moisture content of >20% (43, 59), water and soil with pHs around 5.5 and 7.6 (43, 59–62), and temperatures ranging from 4 to 40°C (36, 60, 63, 64). Likewise, it has been reported that pathogenic Leptospira bacteria can metabolize urea (65) and that they are able to survive for 6 to 18 h in pure (pH 7 to 8) cattle urine, although longer survival times have been observed when urine is diluted (62, 66). Also, the ability of infectious Leptospira bacteria to survive in the environment might be assisted by their ability to resist changes in osmolality (67). Moreover, there is no evidence to suggest that infectious Leptospira bacteria lose their infectivity when they are in the environment and have been shown to remain infectious for at least 43 days in wet soil (61) and 20 months in freshwater (60).

Motility and dispersal of infectious Leptospira bacteria may also be critical for environmental survival by enabling escape from inhospitable microenvironments and tropism toward more favorable conditions. Leptospira bacteria have been shown to move on viscous matrices (around 15 μm/s) and liquid surfaces (5 μm/s) (67). In aqueous environments, chemotaxis toward hemoglobin might lead the pathogen to reach an entrance into the animal body (68). Motility might also facilitate the avoidance of some harmful environmental conditions such as prolonged exposure to sunlight (62, 69, 70).

Additional variables, related to animal reservoirs, might also be important for defining the environmental persistence of Leptospira bacteria in the environment. For instance, the longitudinal survival rate after shedding in urine might depend on urine pH, which is influenced by specific local features such as animal nutrition (71, 72). Abiotic conditions under which infectious Leptospira bacteria survive and persist in the environment have been established mainly from experiments performed under controlled laboratory conditions, and multiple combinations of abiotic conditions have not been tested. Furthermore, these experiments were all qualitative, providing information about the length of survival but not the dynamics of survival over time. There is an unmet need to better comprehend how infectious Leptospira bacteria respond to exposure to a wide diversity of natural soil and water conditions. This information is clearly important for determining the risk of infection from environmental sources.

### Relevance of environmental bacteria.

The composition of microbial communities is also likely to influence the survival and persistence of infectious Leptospira bacteria. While it is probable that some microbial taxa are antagonistic to infectious Leptospira survival in the environment, others might be beneficial. For example, recent research has found that infectious Leptospira bacteria coaggregate with environmental bacteria isolated from freshwater and can live within environmental bacterial biofilms from paddy field water, sewers, and stagnant rainwater (40, 73). While microbial communities may play a very important role in the survival and persistence of infectious Leptospira bacteria in the environment, our knowledge and understanding of this are very limited. More research is needed to further identify, qualify, and quantify the roles of these bacteria in influencing survival, persistence, and ultimately the risk of human/animal infections.

### Genetic factors linked to environmental persistence.

Environmental persistence of pathogenic Leptospira bacteria varies among species (74), but little is known about the genetic mechanisms that drive this difference. From laboratory experiments and whole-genome sequence data, we know that not all Leptospira species have the same ability to survive and reproduce outside a host. For example, after 48 h of incubation in water, Leptospira borgpetersenii serovar Hardjo showed a limited capacity to survive compared to that of Leptospira interrogans (74); however, further details about this observation have not been published. In fact, L. interrogans has been shown to survive and retain virulence in water for up to 344 days (60). The ability of L. interrogans to survive in the environment is consistent with the finding that its genome contains multiple genes (80 in serovar Copenhageni) that code for signal transduction proteins (75) and a higher number of two-component response regulators than L. borgpetersenii (74). Recent research using transposon mutagenesis has provided evidence that the expression of *ebpA*, a gene that encodes an enhancer binding protein that interacts with σ^54^ to activate the transcription of specific genes, is essential for the survival of L. interrogans in freshwater (76). Also, from whole-genome microarray assays, we know that this species has genes that are differentially regulated when it is exposed to environmental temperatures and different osmolality conditions (77, 78). All of these genetic characteristics might play an important role in facilitating the transition of this pathogen between mammalian hosts and soil and water. Additionally, overexpression of the GroEL protein, encoded by *groEl*, has been shown in L. interrogans and Leptospira *fainei* biofilms, suggesting that these protein might have an important role in the persistence of the pathogen in the environment after it is shed in animal urine (79–81).

The scarcity of whole-genome sequences has limited our knowledge of genetic variation within and between Leptospira species. Indeed, half of the whole-genome sequences of the genus in public databases belong to L. interrogans. Fortunately, this situation is changing and between 2014 and 2017, the number of whole-genome sequences increased by about 40%. A major hurdle has been the difficulty in detecting and culturing Leptospira bacteria; however, recent successful experiences in isolating infectious Leptospira bacteria from the environment (38, 82) and new or optimized assays that allow to amplify their DNA and RNA directly from environmental samples with high sensitivity and specificity (17, 39, 40, 83, 84) will help to overcome these limitations. Additionally, genotyping methods have evolved such that now we can better assess the diversity of Leptospira bacteria in a given area or among animal species. For example, older studies have suggested a strong correlation between host species and pathogen type; however, recent work has shown a surprisingly high degree of pathogen diversity in a single reservoir species (13). Furthermore, the use of modern sequencing technologies together with new bioinformatics tools will facilitate the study of the pathogen obtained directly from soil or water, under different conditions, without the need for culturing.

## LINKING LEPTOSPIRA INFECTION TO SPECIFIC ENVIRONMENTAL SOURCES

Despite our knowledge of potential environmental sources and host reservoirs, as well as risk factors for human infection, strong molecular evidence linking individual human cases to specific environmental sources is completely lacking. Evidence that highlights the importance of contact with soil and water as a risk factor for leptospirosis is largely limited to traditional epidemiological surveys that assess exposure to environmental factors. Importantly, molecular evidence that ties a leptospirosis case to a source by establishing contact and a matching genotype has not been found. In many instances, case investigations failed to even detect the pathogen at environmental sites suspected to be the source of outbreaks (56, 57). Nevertheless, there are few examples where the same genotype or serotype was found in water or animal sources from the same community or neighboring sites as a clinical case (13, 17, 28). In one of those studies, clinical samples from the Peruvian Amazon basin were collected from a local hospital while environmental sampling was mainly performed in public areas. Even though a genotype match (at the species level) between an environmental and a clinical sample was found, there was no evidence that patients were in contact with these public areas (28). In another study, clinical samples from the Ecuadorian coast were collected from local health centers while animal samples were extracted from local slaughterhouses. Results were similar to those of the previously cited study; genotype matches were found, but there was no evidence that the clinical cases had direct or indirect contact with the infected animals (13). Another study performed in southern Chile compared clinical samples to those collected from the peridomestic environment of clinical patients but found no genotype matches (17). Unfortunately, to date, there are no studies reporting the detection of infectious Leptospira bacteria from a source where a leptospirosis patient was known (or even likely) to be exposed. This lack of strong evidence that ties individual clinical cases to an environmental source presents a major knowledge gap in our attempts to better understand and identify how humans are infected with Leptospira bacteria.

## CONCLUSION

**FIG 1:**
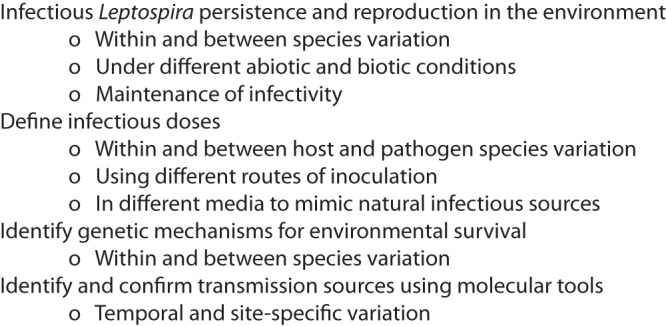
Important aspects to consider for future research.

Leptospira infection most commonly occurs upon exposure to infected animals and contaminated environments; however, filling the critical gaps in our knowledge of the transmission cycle would greatly benefit our efforts to understand the epidemiology of this disease (Fig. 1). To reduce the morbidity rate, we need to better understand how environmental conditions impact Leptospira survival in natural surroundings. What are the characteristics that make some environments more suitable for pathogen persistence? How do different Leptospira species respond to different environmental conditions? What, if any, is the role of environmental bacteria in Leptospira persistence? Can we identify genetic mechanisms that facilitate or restrict survival under certain conditions? Can such genetic characteristics be used as markers for predicting the length of time for which circulating genotypes pose a significant risk of infection? To ascertain the risk of infection, it is extremely important to know the amount of pathogen needed to cause infection in animals and humans. Information about the infectious dose, joined with quantification of the pathogen and knowledge of survival under different environmental conditions, will allow us to make longitudinal predictions of the infection risk posed by exposure to a certain environmental source (Fig. 1).

Leptospirosis has been associated with a wide variety of environmental factors by traditional epidemiological studies. However, routes of exposure to the pathogen are complex and critical differences between sites might exist. Emerging molecular techniques offer the opportunity to better comprehend the relative importance of different potential sources by genotyping the pathogen and matching clinical cases with environmental sources. Although we have much to learn about the relative relevance of each environmental source to human infection, accurate identification of the likelihood of the many different possible sources of infection is critical for understanding the variables associated with environmental exposure.

Understanding temporal and site-specific differences in environmental survival and reproduction of infectious Leptospira bacteria, as well as the likelihood of transmission to humans, will be critical for the development of realistic and effective public health preventive plans. The many possible sources of variation present challenges and opportunities for researchers to incorporate diversity into their laboratory and animal models. Field work in different settings and over longer periods of time will also help identify the relative importance of variables and the extent to which results and conclusions are generalizable across time and space.
